# Supplementary material for: Covalent Triazine Framework C6N6 as an Electrochemical Sensor for Hydrogen-Containing Industrial Pollutants. A DFT Study
Source: Nanomaterials (Basel). 2023 Mar 21;13(6):1121. doi: 10.3390/nano13061121 (PMC10053058; doi:10.3390/nano13061121)
Supplement: Supplementary file 1 [file nanomaterials-13-01121-s001.zip › nanomaterials-2235907-supplementary.pdf]

nanomaterials

MDPI

Supporting Information

Covalent Triazine Framework C_6N_6 as an Electrochemical Sensor for Hydrogen-Containing Industrial Pollutants. A DFT Study

Hassan H. Hammud [1,*,†], Muhammad Yar [2,†], Imene Bayach [1] and Khurshid Ayub [2,*]

[1] Department of Chemistry, College of Science, King Faisal University, Al-Ahsa 31982, Saudi Arabia
[2] Department of Chemistry, COMSATS University Islamabad, Abbottabad Campus, KPK, Islamabad 22060, Pakistan
* Correspondence: hhammoud@kfu.edu.sa (H.H.H.); khurshid@cuiatd.edu.pk (K.A.)
† These authors contributed equally.

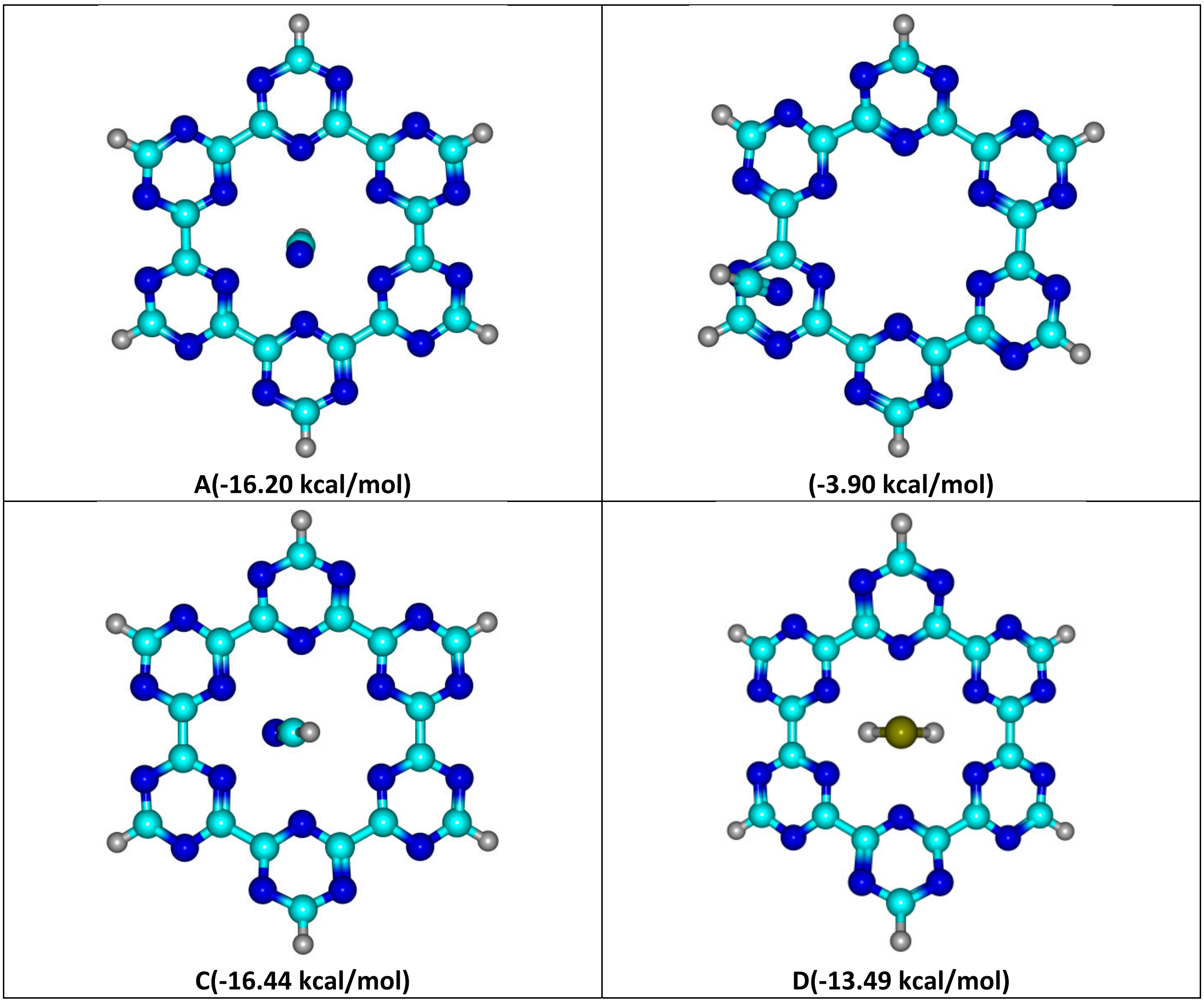

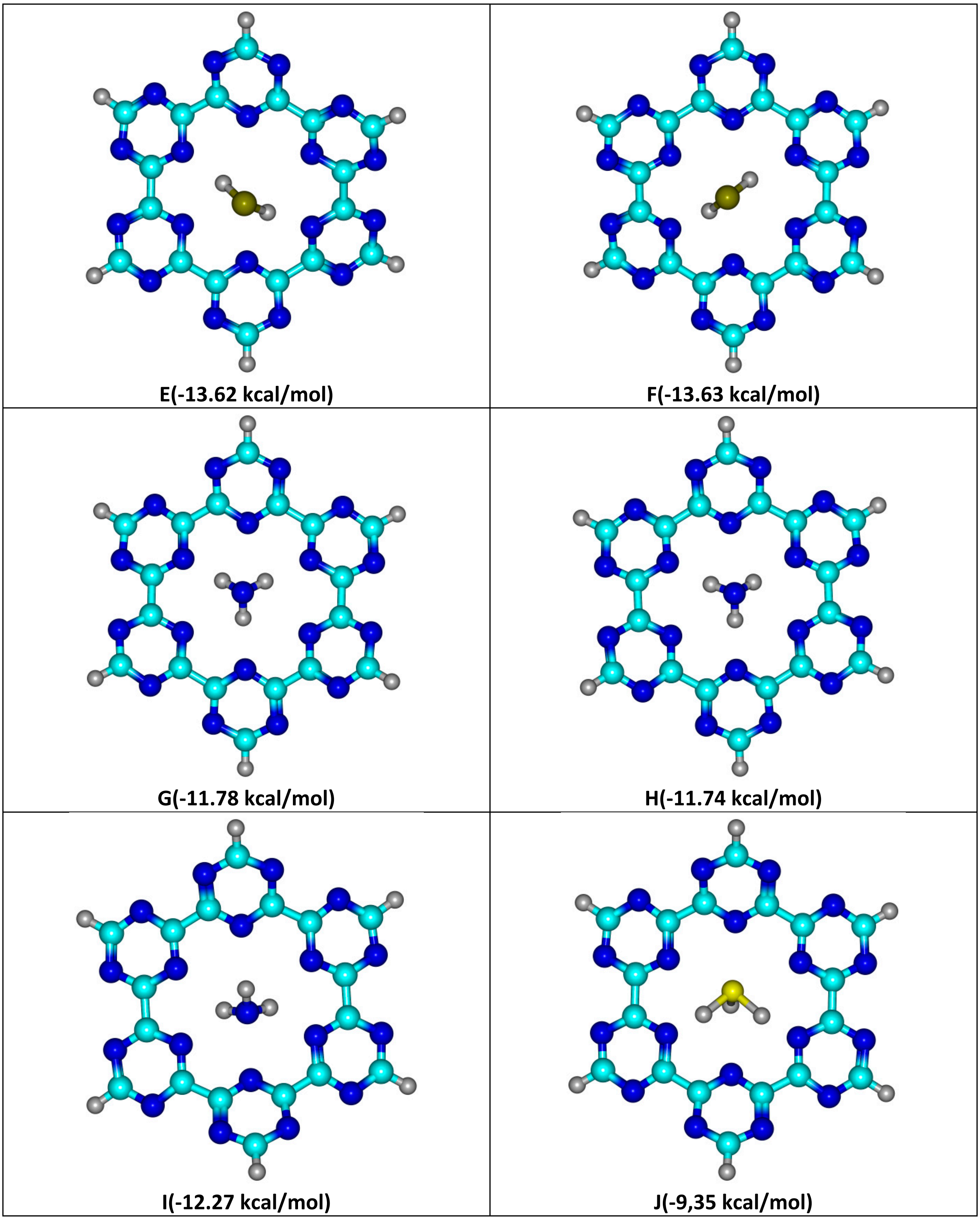

Figure S1. Optimized geometries of least stable complexes of each analyte over C_6N_6 at ωb97XD/6-31G (d,p) level of theory.(HCN/C_6N_6 complexes (A-C), H_2S/C_6N_6 complexes (D-F), NH_3/C_6N_6 complexes (G-I), PH_3/C_6N_6 complexes (J),.
